# A population-based cohort study of fracture risk among 122 255 adults before and after cancer diagnosis compared with matched controls

**DOI:** 10.1093/jbmr/zjag048

**Published:** 2026-03-10

**Authors:** Carrie Ye, William D Leslie, Saeed Al-Azazi, Lin Yan, Lisa M Lix, Piotr Czaykowski, Harminder Singh

**Affiliations:** Department of Medicine, University of Alberta, Edmonton, AB T6G 2G3, Canada; Department of Internal Medicine, University of Manitoba, Winnipeg R3E 0V9, Canada; George and Fay Yee Centre for Healthcare Innovation, University of Manitoba, Winnipeg, R3E0T6, Canada; College of Community and Global Health, University of Manitoba, Winnipeg, R3E 0W3, Canada; College of Community and Global Health, University of Manitoba, Winnipeg, R3E 0W3, Canada; Department of Internal Medicine, University of Manitoba, Winnipeg R3E 0V9, Canada; CancerCare Manitoba, Winnipeg, MB R3E 0V9, Canada; Department of Internal Medicine, University of Manitoba, Winnipeg R3E 0V9, Canada; CancerCare Manitoba, Winnipeg, MB R3E 0V9, Canada

**Keywords:** fracture risk, general population studies, cancer, osteoporosis, epidemiology, survivorship

## Abstract

Individuals with cancer have numerous risk factors for fracture. The objective of this study was to compare fracture risk trajectories in individuals with and without cancer before and after cancer diagnosis. We conducted a matched retrospective cohort study using administrative healthcare and cancer registry data for Manitoba, Canada. We identified residents diagnosed with cancer (excluding nonmelanoma skin cancer) at age ≥18 yr between 1987 and 2014. Each cancer patient was uniquely matched with up to 4 non-cancer individuals based on age, sex, length of residency, and area of residence. Incident clinical fractures were identified using linked healthcare records and validated case definition, before and after cancer diagnosis (index date) up to March 31, 2021. Our cohort comprised 122 255 cancer cases and 460 029 matched controls (average 3.8 non-cancer matches per case). The incident rate of major fracture in the year prior to cancer diagnosis was 10.45 per 1000 person-years (95% CI: 9.77-11.19), increasing to a peak of 20.30 (95% CI: 19.63-21.00) at 1-5 yr post-diagnosis. Incident rate ratios (IRRs) for major, humerus, and vertebral fractures were significantly elevated in cancer vs non-cancer individuals in the year before the index date and remained significantly elevated for up to 5-10 yr. Fracture IRRs were increased across all cancer types except gynecological, melanoma, and thyroid cancers. In conclusion, fracture risk is elevated in the year preceding cancer diagnosis and persists at a higher level compared to the non-cancer population for up to 10 yr. This study emphasizes the necessity of timely and ongoing fracture risk assessment and prompt anti-osteoporosis therapy initiation.

## Introduction

According to the GLOBOCAN estimates, there were approximately 20 million new cancer cases worldwide in 2022^1^ and the global cancer burden is projected to increase, with new cases expected to reach 35 million annually by 2050.^1^ Concurrently, 5-yr survival rates for many cancers have improved globally.^2^ Fracture risk is an important but often under-recognized concern in cancer survivors, significantly decreasing quality of life and even survival.^3^^,^^4^ As advances in cancer treatment continue to improve survival, attention is increasingly turning to the late effects of therapy, including compromised bone health.^5^ Many cancer treatments—such as chemotherapy, hormone therapy, and radiation—can adversely affect bone density and strength, leading to an elevated risk of fractures.^6^ Additionally, factors like aging, reduced physical activity, cancer-induced sarcopenia, and nutritional deficiencies further compound this risk.^7^

Studies have previously reported an increased risk of fractures in several cancer populations. Most studies are restricted to certain cancer subtypes.^8^ Recent studies of a broad range of cancer types have demonstrated increased overall risk of fracture in individuals with cancer, as compared with individuals without cancer.^9–11^ However, these studies are limited by insufficient matching criteria and/or sample size,^9^^,^^11^ lack of evaluation by fracture type^9^ and cancer stage,^9^^,^^10^ or focused only on older adults.^11^ Further, no studies have examined fracture risk in the lead-up period prior to cancer diagnosis.

The objective of this study is to examine time-dependent trajectories in overall and site-specific fracture risk in adult individuals newly diagnosed with cancer, compared to matched individuals without cancer, at short and long-term time intervals before and after cancer diagnosis (index date).

## Materials and methods

### Study design

We performed a matched retrospective cohort study using population-level administrative healthcare data from the province of Manitoba, Canada’s fifth most populous province with a population of 1.48 million.^12^ The healthcare system in Canada is a government-funded single-payer system with free universal access to physician services, hospitals, emergency departments, and laboratory testing.^13^ Each province is the legal custodian of the health data for its citizens. The study was approved by the Health Research Ethics Board for the University of Manitoba [HS18632 (H2015:219)], Manitoba’s Health Information Privacy Committee, and the CancerCare Manitoba Research Resource Impact Committee.

### Data sources

In Manitoba, each resident is assigned a distinct personal health identification number, which allows anonymized linkage of their health care usage and outcomes across provincial administrative databases. The provincial registration database, which lists individuals eligible for health care coverage in Manitoba, provided the demographic data. Cancer data were sourced from the Manitoba Cancer Registry (MCR), which has tracked all cancer cases in the province since 1956. The MCR, a member of both the North American Association of Central Cancer Registries and the Canadian Cancer Registry, is routinely audited to ensure the accurate coding of cancer data.^14^^,^^15^ Its data, including histological confirmation, have been found to be highly comprehensive and accurate.^16^

Data on healthcare visits, hospitalizations, and procedures and their accompanying diagnosis codes were gathered from the Physician Claims Database (PCD) and the Discharge Abstract Database (DAD). The PCD used the International Classification of Diseases, Ninth Revision, Clinical Modification (ICD-9-CM) codes, while the DAD used ICD-9-CM codes until 2004, after which the International Classification of Diseases, Tenth Revision, Canadian Enhancements (ICD-10-CA) was implemented. Medication use was ascertained from the provincial pharmacy database, which records all medications dispensed in the outpatient setting using the World Health Organization’s Anatomic Therapeutic Chemical codes.^17^ Death data was collected from the Vital Statistics Registry, which maintains records of all births and deaths occurring in Manitoba.^18^

### Study population

We identified all Manitoba residents aged 18 yr or older when diagnosed with cancer in the MCR, excluding nonmelanoma skin cancer, between 1987 and 2014. The date of cancer diagnosis and cancer stage at the time of diagnosis were determined as recorded in the MCR. Each individual with cancer was uniquely matched without replacement with up to 4 individuals without a cancer diagnosis based on age (within 5 yr, starting with those closest in birth date), sex, length of residency in Manitoba before the index date (available observation time before diagnosis), and area of residence at the time of cancer diagnosis (nearest neighbor matching method using postal codes).^19^^,^^20^ Unmatched cancer cases were excluded from our analysis. The index date was defined as the date of cancer diagnosis for each cancer case, and this same date was assigned as the index date to their matched control(s).

### Fracture assessment

Incident clinical fractures were identified using the DAD and the PCD based on ICD-9-CM and ICD-10-CA codes up to March 31, 2021. Fracture case definitions used in this study have been previously radiologically validated in fracture and non-fracture cases from the Manitoba BMD Program database and have been adopted for national surveillance.^21^^,^^22^ Briefly, we used site-specific fracture algorithms, requiring that no fracture diagnoses are recorded at the same site within 6 mo prior to the incident fracture date in order to minimize double-counting of the same fracture.^23^ As we were interested in overall fracture burden, pathologic and traumatic fractures were included. Fractures of the hip, forearm, humerus, and clinical vertebral were grouped as major fractures. Hip and forearm fractures were also required to have a site-specific orthopedic procedure code (eg, surgical and casting).^23^ The fracture date was defined as the date of the first clinical encounter for the fracture episode.

### Baseline variables

Specific comorbidity codes, including chronic obstructive pulmonary disease (COPD), rheumatoid arthritis, and alcohol/substance use disorder, were ascertained in the 3 yr prior to the index date. Cancer was not considered when calculating the Charlson comorbidity index. Previous major fracture (any time prior to index date) was assessed using the same algorithms used for incident fracture. We assessed oral glucocorticoid exposure greater than 90 d in the prior year and anti-osteoporosis medication use (alendronate, risedronate, etidronate, raloxifene, calcitonin, zoledronic acid, denosumab, or teriparatide) for at least 180 d in the year prior to the index date. Socio-economic factor index (SEFI) was assessed using methodology that incorporates Canadian Census data with postal codes.^24^ Cancer stage at cancer diagnosis was only available in those diagnosed in 2004 or later. Charlson comorbidity index was assessed using a previously described algorithm.^25^^,^^26^

### Statistical analysis

Summary statistics were used to describe the cancer and non-cancer cohorts. Time of fracture was categorized according to time interval before or after index date (before index date: −10 to <−5 yr, −5 to <−1 yr, and −1 to <0 yr; after index date: 0 to <1 yr, 1 to <5 yr, and 5 to 10 yr). Incidence rate (IR) of each type of fracture was calculated per 1000 person-years of observation time, stratified by time interval, and compared between the cancer and non-cancer cohort to obtain the incident rate ratio (IRR). The 95% CIs for IRRs were calculated using Poisson regression. Incident rate ratios for major fractures were further stratified by sex, age category (<50 yr, 50-70 yr, and >70 yr), and cancer type.

Risk factors for fracture after index date were assessed using multivariable Cox proportional hazards models, which took into account the competing risk of death using the Fine and Gray’s subdistribution hazard model.^27^ Adjusted hazard ratios (aHR) with 95% CIs were reported by cancer type (reference: colorectal cancer), adjusted for sex, age, Charlson comorbidity index, COPD, rheumatoid arthritis, alcohol/substance use disorder, major fracture prior to the index date, socio-economic factor index, and glucocorticoid use. Stage was not included in this multivariable model because stage was only available after 2004. To evaluate the association of cancer stage with major fracture risk, a second multivariable model was evaluated which included cancer stage (stage I/II, stage III/IV, and unknown stage), along with all of the covariates in the preceding model. A *p*-value of <.05 was considered statistically significant. The proportional hazards assumption was confirmed using Schoenfeld residuals. Statistical analyses were conducted using SAS version 9.4 (SAS Institute Inc.).

## Results

There was a total of 127 452 cancer cases initially, of which there were 5197 cancer cases without a match (4.1%), resulting in a total cohort size of 582 284 (122 255 cancer cases, 460 029 non-cancer cases, and average of 3.8 non-cancer matches per cancer case). The non-matched cancer cases were nearly all older females (mean age 79.8 yr, 99.7% female). The matched cancer and non-cancer cohorts were similar in age, sex, and SEFI (Table 1). The cancer cohort had higher Charlson comorbidity index (excluding cancer diagnosis), more COPD and alcohol/substance use disorder diagnoses, and similar rates of prior major fracture, rheumatoid arthritis, and glucocorticoid use. Osteoporotic medication use was low in both groups: 1.6% in the non-cancer group and 1.8% in the cancer group. Of the 48 615 individuals in the cancer cohort diagnosed from 2004 onwards when information on cancer staging became available, 12 777 (26.3%) were stage 1, 11 337 (23.3%) were stage 2, 7641 (15.7%) were stage 3, 9767 (20.1%) were stage 4, and 7093 (14.6%) had unknown stage.

**Table 1:** Cohort description.

	Non-cancer cohort *N* = 460 029	Cancer cohort *N* = 122 255
**Age, years**	63.5 ± 15.6	65.3 ± 15.1
**Male**	210 428 (45.7)	59 776 (48.9)
**Charlson comorbidity index^a^** ** 0** ** 1-2** ** 3-5** ** ≥6**	308 055 (67.0) 129 792 (28.2) 19 045 (4.1) 3148 (0.7)	39 505 (32.3) 52 991 (43.3) 24 095 (19.7) 5664 (4.6)
**Cancer type** ** Colorectal** ** Breast** ** Prostate** ** Lung** ** Urinary tract** ** Hematologic** ** Gynecologic** ** Melanoma** ** Upper GI** ** Head and neck** ** MM/NHL** ** Hepatobiliary** ** Thyroid** ** Others**	n.a.	15 272 (12.5) 17 130 (14.0) 14 658 (12.0) 15 506 (12.7) 6879 (5.6) 4182 (3.4) 8271 (6.8) 9817 (8.0) 3812 (3.1) 3806 (3.1) 6315 (5.2) 3890 (3.2) 1782 (1.5) 10 935 (8.9)
**COPD**	25 269 (5.5)	10 270 (8.4)
**Rheumatoid arthritis**	5583 (1.2)	1444 (1.2)
**Alcohol/substance use disorder**	8494 (1.8)	2787 (2.3)
**Prior major fracture**	32 296 (7.0)	8456 (6.9)
**Osteoporotic medication use**	7528 (1.6)	2209 (1.8)
**Socio-economic factor index quintiles** ** 1 (Highest)** ** 2** ** 3** ** 4** ** 5 (lowest)** ** Missing**	91 716 (19.9) 90 773 (19.7) 89 667 (19.5) 89 137 (19.4) 90 282 (19.6) 8465 (1.8)	23 073 (18.9) 23 610 (19.3) 24 529 (20.1) 25 049 (20.5) 24 135 (19.7) 1859 (1.5)
**Prolonged glucocorticoid use**	3576 (0.8)	1334 (1.1)
**Observation time after index date, years**	10.7 ± 8.4	7.7 ± 8.0
**Death**	200 260 (43.5)	89 891 (73.5)
**Incident major fracture** ** Hip** ** Forearm** ** Humerus** ** Vertebral**	39 932 (8.7) 24 725 (5.4) 23 752 (5.2) 14 186 (3.1) 8485 (1.8)	9356 (7.7) 5294 (4.3) 5839 (4.8) 3625 (3.0) 2357 (1.9)

a Excluding cancer.

All values expressed as mean ± SD or *n*(%).

Abbreviations: COPD, chronic obstructive pulmonary disease; GI, gastrointestinal; MM/NHL, multiple myeloma/non-Hodgkin lymphoma; n.a., not applicable.

The follow-up period (observation time after index date) was shorter in the cancer group (7.7 yr) vs the non-cancer group (10.7 yr). Over the follow-up period, 9356 (7.7%) in the cancer group suffered an incident major fracture, as compared with 39 932 (8.7%) in the non-cancer group. The incident rate per 1000 person-years of major fracture in the cancer cohort was 10.45 (95% CI: 9.77-11.19) in the year prior to cancer diagnosis and increased during each subsequent time interval, peaking at 1 to <5 yr from cancer diagnosis at 20.30 (95% CI: 19.63-21.00), before decreasing back to an incident rate that was similar to the year prior to cancer diagnosis at 5-10 yr post-cancer diagnosis (10.61, 95% CI: 10.11-11.12, Table 2). A similar trend was observed for each of the major fracture subtypes (Table 2). The IRRs of major fracture and specifically humerus and vertebral fractures started to become significantly elevated in the cancer vs non-cancer group in the year prior to the index date (Table 3). The IRRs for all major fractures remained significantly elevated in the cancer group, as compared to the non-cancer group, even 5-10 yr from the index date.

**Table 2:** Fracture incidence per 1000 person years (95% CI), by time interval from index date for cancer cases and controls.

Time interval from index date, years	Major fracture		Hip		Forearm		Humerus		Vertebral	
	Control	Cancer	Control	Cancer	Control	Cancer	Control	Cancer	Control	Cancer
**−10 to <−5**	5.08 (4.96-5.21)	4.92 (4.69-5.16)	0.97 (0.92-1.02)	0.83 (0.74-0.92)	2.05 (1.97-2.13)	2.05 (1.91-2.20)	0.94 (0.94-1.05)	0.94 (0.85-1.04)	1.16 (1.10-1.22)	1.19 (1.07-1.31)
**−5 to <−1**	13.01 (12.77-13.25)	12.70 (12.27-13.14)	3.19 (3.07-3.30)	2.52 (2.35-2.71)	4.69 (4.57-4.82)	4.77 (4.52-5.02)	2.35 (2.35-2.54)	2.55 (2.37-2.74)	2.98 (2.87-3.09)	3.12 (2.90-3.35)
**−1 to <0**	8.29 (7.97-8.63)	10.45 (9.77-11.19)	2.77 (2.59-2.97)	2.74 (2.40-3.13)	2.37 (2.20-2.55)	2.36 (2.04-2.72)	1.32 (1.32-1.59)	1.93 (1.64-2.26)	1.86 (1.71-2.03)	3.75 (3.34-4.20)
**0 to <1**	8.76 (8.42-9.11)	11.95 (11.14-12.82)	3.34 (3.14-3.56)	4.03 (3.57-4.55)	2.19 (2.03-2.37)	2.10 (1.78-2.48)	1.40 (1.40-1.69)	2.35 (2.00-2.75)	1.90 (1.74-2.06)	3.78 (3.34-4.29)
**1 to <5**	17.58 (17.29-17.87)	20.30 (19.63-21.00)	6.36 (6.19-6.53)	6.84 (6.47-7.23)	4.76 (4.63-4.90)	4.97 (4.66-5.31)	3.12 (3.12-3.35)	3.96 (3.69-4.26)	3.70 (3.57-3.83)	5.11 (4.78-5.46)
**5 to 10**	8.67 (8.48-8.86)	10.61 (10.11-11.12)	3.18 (3.07-3.30)	3.68 (3.40-3.98)	2.38 (2.29-2.48)	2.71 (2.47-2.96)	1.49 (1.49-1.65)	2.04 (1.83-2.26)	1.76 (1.68-1.85)	2.55 (2.31-2.81)

**Table 3:** Incidence rate ratio of major fracture and fracture subtypes (95% CI) comparing cancer to control cohorts.

Time interval from index date, years	Major fracture	Hip	Forearm	Humerus	Vertebral
**−10 to <−5**	**0.92 (0.88, 0.97)**	**0.76 (0.69, 0.85)**	0.99 (0.92, 1.06)	0.92 (0.83, 1.02)	0.96 (0.87, 1.06)
**−5 to <−1**	**0.91 (0.88, 0.94)**	**0.68 (0.64, 0.72)**	1.00 (0.95, 1.06)	0.97 (0.91, 1.04)	0.97 (0.91, 1.04)
**−1 to <0**	**1.10 (1.03, 1.17)**	**0.80 (0.71, 0.90)**	0.97 (0.85, 1.11)	**1.20 (1.03, 1.39)**	**1.65 (1.48, 1.85)**
**0 to <1**	**1.15 (1.08, 1.23)**	0.90 (0.81, 1.00)	0.95 (0.82, 1.11)	**1.33 (1.15, 1.55)**	**1.73 (1.53, 1.95)**
**1 to <5**	**1.19 (1.15, 1.23)**	**1.06 (1.01, 1.12)**	**1.11 (1.04, 1.18)**	**1.27 (1.18, 1.37)**	**1.45 (1.36, 1.55)**
**5 to 10**	**1.27 (1.21, 1.34)**	**1.22 (1.13, 1.32)**	**1.17 (1.06, 1.29)**	**1.39 (1.23, 1.56)**	**1.46 (1.31, 1.62)**

Bold font = statistically significant at an alpha error of <.05.

The sex and age-stratified IRRs for major fracture are shown in Figure 1. Males with cancer had higher IRR for major fracture, as compared with females with cancer, at all time intervals (Figure 1A), despite female sex being associated with higher risk of major fracture after cancer diagnosis (aHR 1.76, 95% CI: 1.63-1.90, Table S1). Those individuals with cancer who were <50 yr of age had similar IRR for major fracture as those 50-70 yr of age, and both groups had higher IRRs for major fracture compared with those who were >70 yr of age (Figure 1B). However, increasing age was associated with higher risk of major fracture after cancer diagnosis (aHR per 10-yr increase in age 1.63, 95% CI: 1.60-1.67, Table S1). Other baseline factors associated with fracture after cancer diagnosis include increasing Charlson comorbidity index, COPD, rheumatoid arthritis, alcohol/substance use disorder, prior major fracture, higher SEFI, and glucocorticoid use (Table S1).

**Figure 1:**
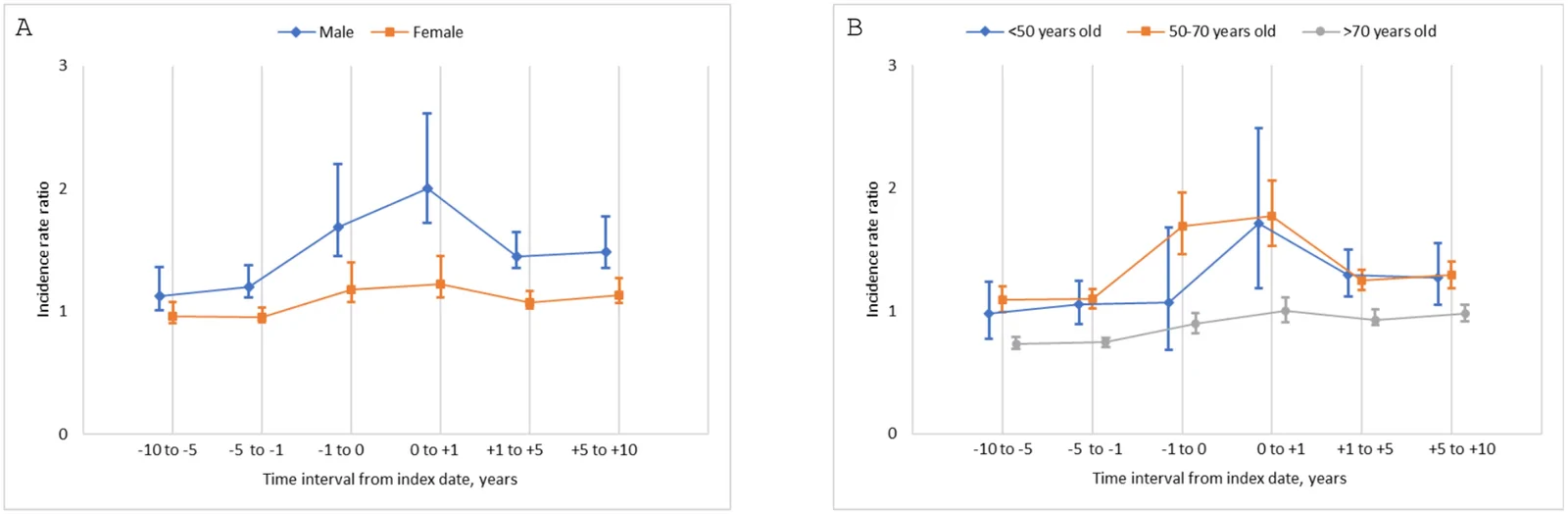
Incident rate ratios (95% confidence bars) for major fracture in cancer group (reference: matched non-cancer) (A) by sex and (B) by age category.

Stratifying results by cancer site showed that the increased IRR for major fracture seen in the cancer group overall appeared to reflect increased risk in nearly all cancer sites, except gynecological, melanoma, and thyroid cancer (Table 4). The highest IRRs were observed with myeloma/non-Hodgkin lymphoma (NHL), which saw a peak IRR of 2.93 (95% CI: 2.39-3.96) in the year prior to diagnosis, 2.36 (95% CI: 1.78-3.12) in the year after diagnosis, and remained elevated at 1.52 (95% CI: 1.23-1.86) out to 5-10 yr. In the cancer cohort, when compared to colorectal cancer as the reference, the risk for major fracture was significantly elevated for hematological (aHR 1.38, 95% CI: 1.18-1.61), lung (aHR 1.32, 95% CI: 1.17-1.50), myeloma/NHL (aHR 1.37, 95% CI: 1.20-1.55), hepatobiliary (aHR 1.46, 95% CI: 1.08-1.97), and other cancers (aHR 1.39, 95% CI: 1.22-1.59) (Table 5). The only cancer type that had a significantly lower aHR for major fracture, as compared with colorectal cancer, was thyroid cancer (aHR 0.73, 95% CI: 0.57-0.95). In the fully adjusted model for major fracture, individuals with stage III/IV cancer had a 13% higher risk of major fracture compared to those with stage I/II (aHR 1.13, 95% CI: 1.03-1.25). Individuals with unknown cancer stage had an even higher fracture risk (aHR 1.27, 95% CI: 1.10-1.48).

**Table 4:** Incidence rate ratio of major fracture by cancer site (reference: matched non-cancer) and time interval from index date.

Cancer site	Time interval from index date (years)					
	−10 to <−5	−5 to <−1	−1 to <0	0 to <1	1 to <5	5 to 10
**Breast**	0.94 (0.83-1.07)	**0.88 (0.80-0.97)**	1.02 (0.83-1.26)	0.96 (0.78-1.18)	**1.19 (1.10-1.28)**	**1.33 (1.20-1.48)**
**Colorectal**	**0.84 (0.73-0.97)**	**0.82 (0.74-0.91)**	0.81 (0.64-1.04)	**1.25 (1.01-1.54)**	**1.13 (1.02-1.24)**	**1.26 (1.10-1.45)**
**Gynecological**	0.96 (0.79-1.17)	0.96 (0.83-1.12)	**0.59 (0.39-0.88)**	0.92 (0.65-1.31)	0.99 (0.87-1.14)	1.02 (0.85-1.23)
**Head/neck**	1.16 (0.84-1.59)	**1.41 (1.12-1.78)**	0.47 (0.21-1.04)	1.31 (0.76-2.23)	**1.43 (1.14-1.79)**	**1.59 (1.16-2.17)**
**Hematological**	0.93 (0.67-1.29)	0.97 (0.77-1.22)	1.28 (0.81-2.02)	**1.60 (1.04-2.47)**	**1.37 (1.14-1.65)**	**1.44 (1.11-1.88)**
**Lung**	1.10 (0.96-1.26)	**1.21 (1.10-1.33)**	**1.86 (1.53-2.25)**	**2.15 (1.75-2.65)**	**1.33 (1.15-1.52)**	**1.55 (1.24-1.93)**
**Melanoma**	0.97 (0.83-1.14)	0.92 (0.83-1.03)	1.05 (0.83-1.34)	1.17 (0.91-1.49)	0.99 (0.88-1.11)	0.96 (0.81-1.14)
**MM/NHL**	1.08 (0.86-1.35)	0.93 (0.79-1.10)	**2.93 (2.29-3.76)**	**2.36 (1.78-3.12)**	**1.45 (1.26-1.68)**	**1.52 (1.23-1.86)**
**Prostate**	1.09 (0.88-1.36)	1.11 (0.95-1.31)	**1.41 (1.01-1.98)**	**1.62 (1.16-2.26)**	**1.59 (1.41-1.79)**	**1.50 (1.27-1.76)**
**Hepatobiliary**	1.12 (0.87-1.44)	1.05 (0.87-1.26)	1.43 (0.98-2.08)	**2.09 (1.33-3.30)**	1.05 (0.68-1.63)	1.60 (0.76-3.38)
**Thyroid**	1.08 (0.61-1.91)	1.01 (0.65-1.59)	1.07 (0.30-3.85)	0.42 (0.10-1.81)	0.93 (0.64-1.36)	1.04 (0.64-1.68)
**Upper GI**	0.75 (0.54-1.05)	0.83 (0.66-1.05)	0.89 (0.55-1.44)	1.52 (0.94-2.45)	1.19 (0.87-1.61)	**1.71 (1.12-2.61)**
**Urinary tract**	1.03 (0.79-1.35)	1.06 (0.88-1.27)	1.06 (0.73-1.55)	**1.76 (1.26-2.46)**	**1.24 (1.05-1.47)**	**1.44 (1.15-1.81)**
**Others**	0.87 (0.73-1.04)	1.02 (0.91-1.15)	**1.78 (1.40-2.26)**	**1.40 (1.06-1.85)**	1.03 (0.89-1.19)	1.02 (0.82-1.27)

Bold font = statistically significant at an alpha error of <.05.

Abbreviations: GI, gastrointestinal; MM/NHL, multiple myeloma/non-Hodgkin lymphoma.

**Table 5:** Adjusted hazard ratios with 95% CIs for major fracture after the index date in the cancer cohort, by cancer type.

Cancer type	Adjusted^a^ HR (95% CI) for major fracture
	Full cancer cohort *N* = 122 255
**Colorectal**	Reference
**Breast**	1.02 (0.93, 1.13)
**Gynecological**	0.90 (0.79, 1.01)
**Head/neck**	1.11 (0.93, 1.33)
**Hematological**	**1.38 (1.18, 1.61)**
**Lung**	**1.32 (1.17, 1.50)**
**Melanoma**	1.00 (0.90, 1.12)
**MM/NHL**	**1.37 (1.20, 1.55)**
**Prostate**	0.98 (0.88, 1.10)
**Hepatobiliary**	**1.46 (1.08, 1.97)**
**Thyroid**	**0.73 (0.57, 0.95)**
**Upper GI**	1.19 (0.94, 1.51)
**Urinary tract**	1.04 (0.90, 1.19)
**Others**	**1.39 (1.22, 1.59)**

a Adjusted for sex, age, Charlson comorbidity index, chronic obstructive pulmonary disease, rheumatoid arthritis, alcohol/substance use disorder, prior major fracture, socio-economic factor index, and glucocorticoid use.

Bold font = statistically significant at an alpha error of <.05.

Abbreviations: GI, gastrointestinal; MM/NHL, multiple myeloma/non-Hodgkin lymphoma.

## Discussion

Fracture risk increased starting in the year prior to cancer diagnosis, and generally remained elevated even 5-10 yr after cancer diagnosis, peaking at around 20 major fractures per 1000 person years in the 1-5 yr after cancer diagnosis, which was about 20% higher than in those without cancer. In the first year after cancer diagnosis, the incidence of major fracture was 15% higher, and of vertebral fractures, 73% higher, than in those without cancer. Individuals with hematologic, lung, melanoma, myeloma/NHL, and hepatobiliary cancer, as well as those with stage III and IV cancers, were at particularly higher risk of fracture. Interestingly, while fracture risk was elevated after cancer diagnosis for nearly all cancer types, melanoma and thyroid cancer were not associated with increased fracture risk at any time period before or after cancer diagnosis, as compared to the matched non-cancer cases. Males and those younger than 70 yr of age with cancer were at relatively greater fracture risk than their matched non-cancer controls, likely reflecting the low absolute fracture risk in the controls, consistent with female sex and older age being risk factors for fracture (Table S1).

This study confirms previous studies that fracture risk increases after cancer diagnosis.^9–11^ The etiology for increased fracture risk in the cancer population is likely multifactorial.^8^ Previous studies have shown that patients with cancer have a high prevalence of osteoporosis.^28–30^ However, it is important to note that BMD testing is very low in the cancer population,^4^ so BMD data from clinical cancer cohorts are subject to selection bias. Similarly, osteoporosis treatment rates are low in the cancer population,^31^ as confirmed in our study, which showed that 6.9% of the cancer cohort had a prior major fracture, but only 1.8% of this cohort were on anti-osteoporotic medications at the time of cancer diagnosis.

Strengths of this population-level study include the large sample size and long observation period before and after cancer diagnosis. This, along with matching based on observation period prior to cancer diagnosis, allowed us to examine the trajectory in fracture risk up to 10 yr prior to and after cancer diagnosis. Unlike previous studies, which have shown an increase in fracture risk after cancer diagnosis, we found that fracture risk, especially for hip fracture, is actually lower 1-10 yr prior to cancer diagnosis, but is increased by about 10% in the year leading up to a cancer diagnosis, compared to the matched non-cancer cohort. Thus fracture risk increases shortly before the date of cancer diagnosis and may actually be an early sign of cancer and/or lead to detection of cancer. The relatively lower risk of fracture in the cancer group up to the year before cancer diagnosis may be due to factors such as obesity, which is generally associated with higher BMD,^32^ but associated with increased cancer risk.^33^ In fact, high BMD has even been identified as a risk factor for breast cancer.^34^^,^^35^

Additionally, unlike most previous studies which did not have cancer stage data, we were able to show that individuals with late-stage cancer were at 13% greater risk of fracture as compared with those with early-stage cancer. This finding should prompt greater consideration of anti-osteoporosis therapy in patients with late-stage cancer. Further, by matching on area of residency, along with adjusting for socio-economic factor index, we were able to partially account for differences in access to care and healthcare utilization between groups. We considered the competing risk of death in our time to fracture analysis, an important real-world consideration when examining cancer populations. Finally, we used site-specific fracture case definitions, which have been previously validated in our population.^22^

We acknowledge that our study had limitations. The unmatched cancer cases were older than the matched cancer cases, and nearly all were older females, potentially resulting in some selection bias with older females being under-represented. We did not have data on BMI or smoking status, although we adjusted for COPD diagnosis as a proxy of smoking, recognizing that this is not a perfect surrogate. Further adjustment for these variables may provide additional refinement of our estimates as BMI and smoking are both associated with cancer and fracture risk. While fractures were ascertained in the same way for both the cancer and non-cancer cohorts, there is a risk of outcome-detection bias if individuals in the cancer cohort had more healthcare utilization, and in particular more frequent imaging, which may have increased the likelihood of diagnosing fractures. However, this is most likely to bias the detection of non-clinical vertebral compression fractures, and our study found that the IRRs of all major fractures including non-vertebral fractures were increased in the cancer cohort. We chose not to distinguish between pathologic and osteoporotic fractures because previous studies have shown that there is a high risk of misclassification between these categories in administrative data.^36^ Finally, we did not have data on cancer treatment, or specific bone metastasis data, which are likely effect modifiers of fracture risk in cancer and would have made for interesting stratified analyses. Similarly, we did not have data on ethnicity, which may also be an effect modifier, although a previous study showed little variation of fracture risk by ethnicity in cancer survivors.^10^

This study underscores the importance of timely and continued fracture risk assessment and early initiation of anti-osteoporosis therapy. Not only is fracture risk increased after cancer diagnosis, fracture risk increases in the year prior to cancer diagnosis, and yet very few individuals are on anti-osteoporosis therapy during this time period. In these individuals, it is especially important that anti-osteoporosis medications be initiated prior to cancer therapies, as many cancer therapies, including radiation, chemotherapy, hormonal therapies, and glucocorticoids, are known to adversely impact on bone health and further increase the risk of fractures.^6^^,^^11^^,^^37^ Importantly, this increased risk of fracture persists long after the initial cancer diagnosis, and continues to put individuals at higher risk of major fracture even 10 yr after cancer diagnosis. This calls for long-term vigilance and attention to bone health in cancer survivors. Fortunately, existing fracture risk calculators, such as FRAX, predict 10-yr fracture risk in individuals with cancer.^38^ With the recent rapid adoption of immunotherapy as the newest pillar in cancer treatment,^39^ and evidence that these therapies may have detrimental effects on bone,^40–43^ continued surveillance of fracture risk in cancer populations is needed as therapies rapidly shift towards immune-based treatments.

## Supplementary Material

Supplemental_Materials_(1)_zjag048

## Data Availability

Data sharing is not permitted under the Researcher Agreement with Manitoba Health and Seniors Care (MHASC). However, researchers may apply for data access through the Health Research Ethics Board for the University of Manitoba and the Health Information and Privacy Committee of MHASC.
