# Supplementary material for: A population-based cohort study of fracture risk among 122 255 adults before and after cancer diagnosis compared with matched controls
Source: J Bone Miner Res. 2026 Mar 10;41(11):1232–40. doi: 10.1093/jbmr/zjag048 (PMC13716916; doi:10.1093/jbmr/zjag048)
Supplement: Supplementary file 1 [file supplemental_materials_(1)_zjag048.docx]

**Supplementary Materials**

**eTable 1. Adjusted* hazard ratio (95% confidence interval) for major fracture after the index date in the cancer cohort**

|  | Major fracture | Hip | Forearm | Humerus | Vertebral |
| --- | --- | --- | --- | --- | --- |
| Female | **1.76 (1.63-1.90)** | **1.56 (1.38-1.76)** | **3.10 (2.61-3.67)** | **2.09 (1.76-2.48)** | 1.08 (0.95-1.24) |
| Age, per 10-year increase | **1.63 (1.60-1.67)** | **2.75 (2.63-2.88)** | **1.14 (1.09-1.18)** | **1.48 (1.41-1.55)** | **1.53 (1.46-1.60)** |
| CCI  0  1  2  ≥3 | Reference  **1.13 (1.04-1.23)**  **1.12 (1.05-1.19)**  **1.27 (1.17-1.37)** | Reference  **1.25 (1.09-1.42)**  **1.13 (1.01-1.26)**  **1.40 (1.23-1.58)** | Reference  0.92 (0.78-1.10)  1.12 (0.99-1.27)  1.12 (0.95-1.32) | Reference  **1.20 (1.01-1.43)**  0.99 (0.85-1.14)  1.17 (0.98-1.39) | **Reference**  **1.21 (1.03-1.43)**  **1.27 (1.12-1.45)**  **1.42 (1.22-1.66)** |
| COPD | 1.12 (1.00-1.25) | 1.10 (0.93-1.30) | 1.15 (0.89-1.48) | 1.09 (0.85-1.39) | 1.17 (0.96-1.44) |
| Rheumatoid arthritis | **1.40 (1.15-1.70)** | 1.34 (0.98-1.85) | 1.28 (0.85-1.95) | **1.80 (1.24-2.60)** | 1.07 (0.71-1.62) |
| Alcohol/substance use disorder | **1.51 (1.23-1.86)** | **2.19 (1.59-3.01)** | 1.27 (0.82-1.97) | 1.50 (0.96-2.34) | 1.26 (0.83-1.90) |
| Prior major fracture | **2.04 (1.89-2.20)** | **1.63 (1.45-1.84)** | **2.36 (2.03-2.74)** | **2.16 (1.84-2.53)** | **2.26 (1.96-2.61)** |
| SEFI quintile  1 (lowest)  2  3  4  5  missing | Reference  1.06 (0.97-1.15)  1.02 (0.93-1.11)  1.05 (0.97-1.15)  **1.14 (1.05-1.24)**  **1.82 (1.32-2.50)** | Reference  1.02 (0.89-1.17)  1.02 (0.88-1.17)  1.06 (0.93-1.21)  1.12 (0.97-1.29)  **3.04 (2.00-4.65)** | Reference  0.92 (0.79-1.09)  0.95 (0.81-1.12)  0.91 (0.77-1.07)  1.08 (0.92-1.28)  0.50 (0.16-1.57) | Reference  1.19 (0.99-1.43)  1.10 (0.91-1.32)  1.11 (0.92-1.33)  **1.25 (1.03-1.51)**  **2.82 (1.57-5.05)** | Reference  **1.21 (1.03-1.43)**  1.07 (0.90-1.27)  **1.19 (1.01-1.40)**  **1.20 (1.01-1.42)**  0.90 (0.37-2.18) |
| Glucocorticoid use | **1.54 (1.26-1.89)** | **1.58 (1.15-2.17)** | 1.36 (0.86-2.17) | 1.33 (0.84-2.11) | **1.70 (1.18-2.46)** |
| Cancer type  Colorectal  Breast  Gynecological  Head / Neck  Hematological  Lung  Melanoma  MM/NHL  Prostate  Thyroid  Upper GI  Urinary Tract  Others | Reference  1.02 (0.93-1.12)  0.90 (0.79-1.01)  1.11 (0.93-1.33)  **1.38 (1.18-1.61)**  **1.32 (1.17-1.50)**  1.00 (0.90-1.12)  **1.37 (1.20-1.55)**  0.98 (0.87-1.10)  0.73 (0.57-0.95)  1.19 (0.94-1.51)  1.03 (0.90-1.19)  **1.39 (1.22-1.59)** | Reference  0.91 (0.79-1.06)  0.88 (0.72-1.09)  **1.32 (1.02-1.73)**  **1.34 (1.05-1.72)**  **1.57 (1.30-1.90)**  0.94 (0.79-1.11)  1.17 (0.94-1.45)  1.12 (0.94-1.34)  0.64 (0.37-1.12)  1.39 (0.98-1.98)  0.94 (0.75-1.19)  **1.40 (1.14-1.73)** | Reference  1.09 (0.91-1.30)  0.81 (0.64-1.01)  1.08 (0.75-1.55)  1.09 (0.80-1.49)  0.92 (0.68-1.22)  0.96 (0.76-1.21)  1.03 (0.78-1.36)  1.02 (0.78-1.33)  0.66 (0.44-1.00)  0.95 (0.55-1.64)  1.04 (0.79-1.38)  1.13 (0.86-1.48) | Reference  0.98 (0.80-1.19)  1.02 (0.80-1.30)  0.59 (0.36-0.96)  0.85 (0.58-1.26)  1.15 (0.87-1.52)  0.84 (0.66-1.07)  1.23 (0.93-1.63)  0.86 (0.65-1.12)  0.58 (0.33-1.02)  1.12 (0.67-1.86)  1.08 (0.80-1.45)  **1.37 (1.04-1.80)** | Reference  1.09 (0.90-1.32)  0.89 (0.68-1.16)  1.24 (0.89-1.72)  **1.85 (1.41-2.43)**  **1.36 (1.06-1.73)**  1.06 (0.86-1.32)  **2.04 (1.63-2.55)**  0.94 (0.76-1.17)  0.83 (0.50-1.40)  1.03 (0.63-1.67)  1.11 (0.86-1.45)  **1.52 (1.19-1.95)** |

**Cox regression model adjusted for all variables listed in this table. Bold font=statistically significant at a nominal alpha of <0.05. CCI=Charlson comorbidity index, COPD=chronic obstructive pulmonary disease, SEFI=socio-economic factor index, MM/NHL=Multiple myeloma/non-Hodgkin lymphoma, GI=gastrointestinal.*
